# Elastic Properties and Energy Loss Related to the Disorder–Order Ferroelectric Transitions in Multiferroic Metal–Organic Frameworks [NH_4_][Mg(HCOO)_3_] and [(CH_3_)_2_NH_2_][Mg(HCOO)_3_]

**DOI:** 10.3390/ma14113125

**Published:** 2021-06-07

**Authors:** Zhiying Zhang, Hongliang Yu, Xin Shen, Lei Sun, Shumin Yue, Hao Tang

**Affiliations:** School of Materials Science and Engineering, Wuhan University of Technology, Wuhan 430070, China; yuhongliang2021@126.com (H.Y.); shenxin1221@126.com (X.S.); sunlei15171@163.com (L.S.); yueshumin1216@163.com (S.Y.); tanghao202103@163.com (H.T.)

**Keywords:** metal–organic framework (MOF), ferroelectric transition, dynamic mechanical analysis (DMA), elastic property, energy loss

## Abstract

Elastic properties are important mechanical properties which are dependent on the structure, and the coupling of ferroelasticity with ferroelectricity and ferromagnetism is vital for the development of multiferroic metal–organic frameworks (MOFs). The elastic properties and energy loss related to the disorder–order ferroelectric transition in [NH_4_][Mg(HCOO)_3_] and [(CH_3_)_2_NH_2_][Mg(HCOO)_3_] were investigated using differential scanning calorimetry (DSC) and dynamic mechanical analysis (DMA). The DSC curves of [NH_4_][Mg(HCOO)_3_] and [(CH_3_)_2_NH_2_][Mg(HCOO)_3_] exhibited anomalies near 256 K and 264 K, respectively. The DMA results illustrated the minimum in the storage modulus and normalized storage modulus, and the maximum in the loss modulus, normalized loss modulus and loss factor near the ferroelectric transition temperatures of 256 K and 264 K, respectively. Much narrower peaks of loss modulus, normalized loss modulus and loss factor were observed in [(CH_3_)_2_NH_2_][Mg(HCOO)_3_] with the peak temperature independent of frequency, and the peak height was smaller at a higher frequency, indicating the features of first-order transition. Elastic anomalies and energy loss in [NH_4_][Mg(HCOO)_3_] near 256 K are due to the second-order paraelectric to ferroelectric phase transition triggered by the disorder–order transition of the ammonium cations and their displacement within the framework channels, accompanied by the structural phase transition from the non-polar hexagonal *P*6_3_22 to polar hexagonal *P*6_3_. Elastic anomalies and energy loss in [(CH_3_)_2_NH_2_][Mg(HCOO)_3_] near 264 K are due to the first-order paraelectric to ferroelectric phase transitions triggered by the disorder–order transitions of alkylammonium cations located in the framework cavities, accompanied by the structural phase transition from rhombohedral *R*$\bar{3}$c to monoclinic *C*c. The elastic anomalies in [NH_4_][Mg(HCOO)_3_] and [(CH_3_)_2_NH_2_][Mg(HCOO)_3_] showed strong coupling of ferroelasticity with ferroelectricity.

## 1. Introduction

Metal–organic frameworks (MOFs) have attracted intensive attention due to their tunable properties and potential applications in gas storage and separations, catalysis, photoluminescence, sensors, magnetic and electric applications [1,2,3,4,5,6,7]. Multiferroic MOFs with at least two coexisting orders among the ferroelectricity, ferromagnetism and ferroelasticity are of particular interest, and their structure analysis, thermal properties, electric properties and magnetic properties have been widely studied using X-ray diffraction (XRD), neutron scattering, infrared spectroscopy, Raman spectroscopy, differential scanning calorimetry (DSC), heat capacity measurements, dielectric measurements, magnetic measurements and computer simulations [8,9,10,11,12,13,14].

In [NH_4_][M(HCOO)_3_] (M = Mg, Mn, Fe, Co, Ni, Zn), paraelectric to ferroelectric phase transitions triggered by the disorder–order transitions of the ammonium cations and their displacement within the framework channels occur between 191 K and 255 K (Co: 191 K, Zn: 192 K, Ni: 199 K, Fe: 212 K, Mn: 254 K, Mg: 255 K), accompanied by the structural phase transition from the non-polar hexagonal space group *P*6_3_22 to the polar hexagonal space group *P*6_3_ [15,16,17,18,19,20,21,22,23,24,25,26,27]. Moreover, in [NH_4_][M(HCOO)_3_] (M = Mn, Fe, Co, Ni), spin-canted antiferromagnetic ordering (M = Mn, Co, Ni) or ferromagnetic ordering (M = Fe) occurs between 8 K and 30 K (Mn: 8.4 K, Fe: 9.4 K, Co: 9.8 K, Ni: 29.5 K), indicating the existence of magnetoelectric coupling [20,21,22,23,24,25,26,27]. According to first-principle calculations, it is predicted that magnetism and ferroelectricity also coexist in [NH_4_][M(HCOO)_3_] (M = Sc, Ti, V, Cr, Cu) [28]. 

In [(CH_3_)_2_NH_2_][M(HCOO)_3_] (M = Mg, Mn, Fe, Co, Ni, Zn), the paraelectric–ferroelectric phase transitions triggered by the order–disorder of the alkylammonium cations located in the framework cavities occur at 156 K–267 K (Zn: 156 K, Fe: 160 K, Co: 165 K, Ni: 180 K, Mn: 185 K, Mg: 267 K), associated with a structural phase transition from rhombohedral *R*$\bar{3}$c to monoclinic *Cc* [29,30,31,32,33,34,35,36,37,38,39,40,41]. The ferroelectric transition temperature is the highest when M is Mg, because Mg^2+^ is the hardest Lewis acid among them, leading to the strongest hydrogen bonds [35]. Furthermore, the spin-canted antiferromagnetic ordering transitions in [(CH_3_)_2_NH_2_][M(HCOO)_3_] (M = Mn, Co, Ni, Fe, Cu) occur below 40 K (Ni: 35.6 K, Fe: 20 K, Co: 14.9 K, Mn: 8.5 K, Cu: 5.2 K) due to the metal ions within the framework skeletons, and the spin reorientation transitions in [(CH_3_)_2_NH_2_][M(HCOO)_3_] (M = Co, Ni) occur at 13.1–14.3 K (Co: 13.1 K, Ni: 14.3 K) [42,43,44,45,46,47,48,49,50,51,52,53]. The effect of pressure on the structure and the electric and magnetic properties of [(CH_3_)_2_NH_2_][M(HCOO)_3_] (M = Mg, Mn, Co, Zn, Cd) was investigated [37,47,54,55,56].

The ferroelectric transition temperatures of [NH_4_][Mg(HCOO)_3_] and [(CH_3_)_2_NH_2_][Mg(HCOO)_3_] are around 255 K and 267 K, respectively, close to room temperature, leading to promising applications. Their synthesis, structure analysis, thermal properties, dielectric properties, IR studies and Raman studies have been well studied [13,14,15,33,34,35,36,37,38,57,58]. However, their elastic properties have not been reported. The present work studied the elastic properties and energy loss of [NH_4_][Mg(HCOO)_3_] and [(CH_3_)_2_NH_2_][Mg(HCOO)_3_] associated with ferroelectric transitions using dynamic mechanical analysis (DMA) at low frequencies of 0.1–10 Hz and at high stress and strain levels and investigated the relationship between the structure of MOFs and their elastic properties. The findings quantified the changes in elastic properties and energy loss related to the ferroelectric phase transition and contributed to the further understanding of the phase transition mechanism.

## 2. Materials and Methods

### 2.1. Material Synthesis

Single crystals of [NH_4_][Mg(HCOO)_3_] and [(CH_3_)_2_NH_2_][Mg(HCOO)_3_] were synthesized by the slow diffusion methods described in [15] and [37], respectively. To obtain crystals of [NH_4_][Mg(HCOO)_3_], a 16 mL methanol solution containing 12.8 mmol [NH_4_][HCOO] and 12.8 mmol HCOOH was placed at the bottom of a tube, and then a 16 mL methanol solution containing 1.6 mmol of MgCl_2_ was gently added. The tube was sealed and kept undisturbed, and colorless crystals of [NH_4_][Mg(HCOO)_3_] were harvested after 1 week. To obtain crystals of [(CH_3_)_2_NH_2_][Mg(HCOO)_3_], a 10 mL methanol solution containing 6 mmol of dimethylamine and 8 mmol of formic acid was placed at the bottom of a tube, and then a 16 mL methanol solution containing 2 mmol MgCl_2_ was gently added. The tube was sealed and kept undisturbed, and colorless crystals of [(CH_3_)_2_NH_2_][Mg(HCOO)_3_] were harvested after 1 week. The crystals were filtered from the mother liquid and washed in ethanol 5 times.

### 2.2. Morphology

The morphology of the [NH_4_][Mg(HCOO)_3_] and [(CH_3_)_2_NH_2_][Mg(HCOO)_3_] crystals were examined by SH11/YF9 (ZhongXi, Beijing, China) optical microscopy (OM) and JSM-IT300 (JOEL, Tokyo, Japan) scanning electron microscopy (SEM).

### 2.3. Powder XRD

The powder XRD patterns of [NH_4_][Mg(HCOO)_3_] and [(CH_3_)_2_NH_2_][Mg(HCOO)_3_] were collected through a Bruker D8 Advance diffractometer (Bruker, Billerica, MA, USA) using Cu Kα radiation with a wavelength of 1.5406 Å at 40 kV and 40 mA. The diffraction angle 2 was in the range of 10–60° and the step size was 0.02°. The Rietveld fit of the XRD patterns was obtained using GSAS.

### 2.4. DSC

The DSC measurements of [NH_4_][Mg(HCOO)_3_] and [(CH_3_)_2_NH_2_][Mg(HCOO)_3_] (around 10 mg) were performed through Netzsch DSC 200F3 (Netzsch, Selb, Germany) in the range of 200–300 K during cooling and heating processes at the rate of 5 K/min.

### 2.5. DMA

DMA measurements of the single crystals of [NH_4_][Mg(HCOO)_3_] and [(CH_3_)_2_NH_2_][Mg(HCOO)_3_] were carried out using DMA8000 instruments (PerkinElmer Instruments, Waltham, MA, USA) in the single cantilever mode from 160 K to 320 K at the rate of 2 K/min. DMA measurements of pellets of [NH_4_][Mg(HCOO)_3_] and [(CH_3_)_2_NH_2_][Mg(HCOO)_3_] were carried out in the compression mode using PerkinElmer Instruments Diamond DMA (PerkinElmer Instruments, Waltham, MA, USA) in the range of 140–300 K at 2 K/min during heating processes, as described in [39,53].

## 3. Results and Discussion

### 3.1. Morphology

**Figure 1:**
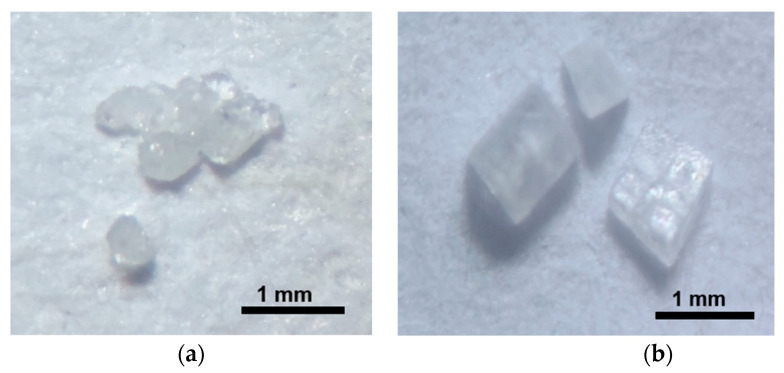
OM image of (**a**) [NH_4_][Mg(HCOO)_3_] and (**b**) [(CH_3_)_2_NH_2_][Mg(HCOO)_3_] crystals.

**Figure 2:**
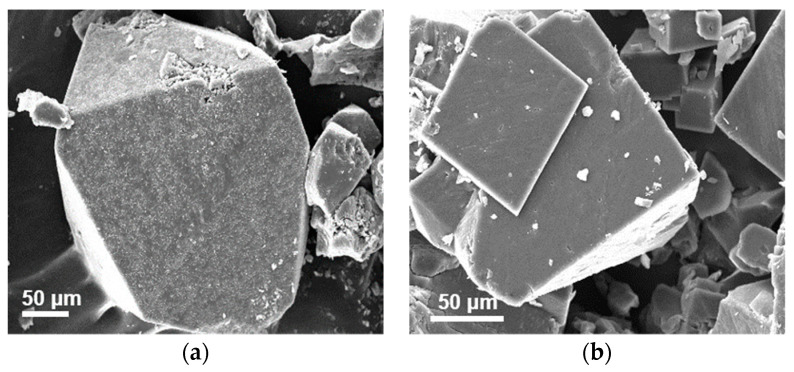
SEM image of (**a**) [NH_4_][Mg(HCOO)_3_] and (**b**) [(CH_3_)_2_NH_2_][Mg(HCOO)_3_] crystals.

The OM and SEM images of [NH_4_][Mg(HCOO)_3_] and [(CH_3_)_2_NH_2_][Mg(HCOO)_3_] crystals are illustrated in Figure 1 and Figure 2. The sizes of [NH_4_][Mg(HCOO)_3_] and [(CH_3_)_2_NH_2_][Mg(HCOO)_3_] crystals are around 0.2 mm and 0.5 mm, respectively.

### 3.2. Powder XRD

**Figure 3:**
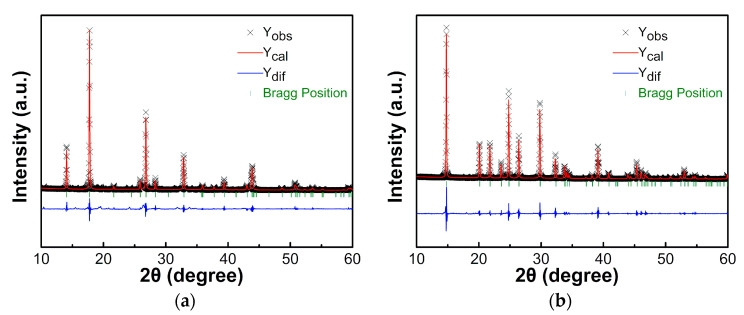
Rietveld fit of XRD patterns of (**a**) [NH_4_][Mg(HCOO)_3_] and (**b**) [(CH_3_)_2_NH_2_][Mg(HCOO)_3_] at room temperature.

The experimental powder XRD pattern of [NH_4_][Mg(HCOO)_3_] at room temperature is consistent with the simulated XRD pattern of the non-polar hexagonal space group *P*6_3_22 (CCDC 949604), as demonstrated in Figure 3a. The lattice parameters obtained by Rietveld refinement are a = b = 7.2729(3) Å, c = 8.2104(5) Å, α = β = 90°, γ = 120°, *R*_wp_ = 15.53% and *R*_p_ = 11.38%. These results agree with the results reported by Maczka et al. [15], which were a = b = 7.8230(10) Å, c = 8.2240(16) Å, α = β = 90° and γ = 120°.

The experimental powder XRD pattern of [(CH_3_)_2_NH_2_][Mg(HCOO)_3_] at room temperature is consistent with the simulated XRD pattern of the space group rhombohedral *R*$\bar{3}$c (CCDC 696736), as shown in Figure 3b. The refined lattice parameters are a = b = 8.1525(1) Å, c = 22.6015(6) Å, α = β = 90°, γ = 120°, *R*_wp_ = 11.69% and *R*_p_ = 9.09%. These are consistent with the results reported by Rossin et al. [33] which were a = b = 8.149(3) Å, c = 22.598(3) Å, *R*_wp_ = 4.71% and *R*_p_ = 12.03%. 

**Figure 4:**
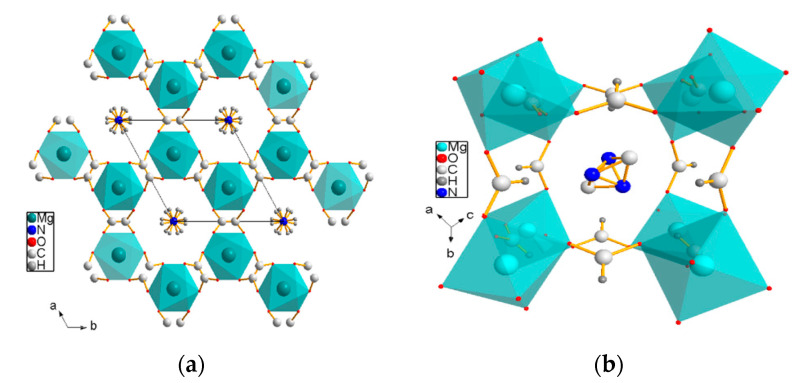
Structures of (**a**) [NH_4_][Mg(HCOO)_3_] and (**b**) [(CH_3_)_2_NH_2_][Mg(HCOO)_3_] at room temperature.

The structures of [NH_4_][Mg(HCOO)_3_] and [(CH_3_)_2_NH_2_][Mg(HCOO)_3_] at room temperature are demonstrated in Figure 4.

### 3.3. DSC

**Figure 5:**
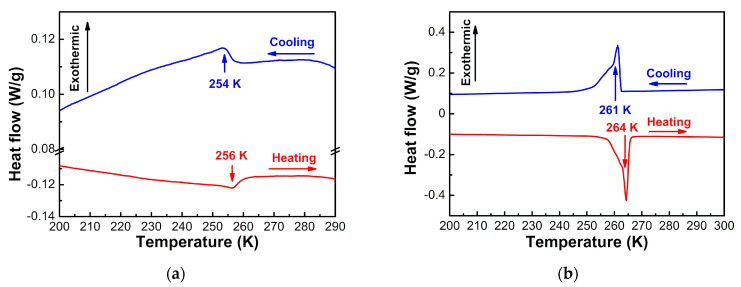
DSC curves of (**a**) [NH_4_][Mg(HCOO)_3_] and (**b**) [(CH_3_)_2_NH_2_][Mg(HCOO)_3_] during cooling and heating processes at the rate of 5 K/min.

Figure 5a displays the DSC curves of [NH_4_][Mg(HCOO)_3_] with anomalies at 254–256 K at the cooling and heating rate of 5 K/min. This agrees with the transition temperature of 254–255 K reported by Maczka et al. at the rate of 15 K/min [15]. The enthalpy ∆H was determined to be 53.0 – 62.4 J·mol^−1^ and the entropy ΔS was determined to be 0.246–0.207 J·mol^−1^·K^−1^. The ratio of the number of configurations in the disordered and ordered systems, N, was determined to be 1.025–1.030. It suggests that the transition is more complex than a simple 3-fold order–disorder model, with N around 3. Maczka et al. reported that the λ-type anomaly in the DSC curve indicated that the ferroelectric transition in [NH_4_][Mg(HCOO)_3_] was second order [15]. Variable-temperature Raman spectra indicated no obvious jumps in the characteristic peak positions of the vibration groups near the transition temperature, indicating the nature of the second-order phase transition [19,22].

Figure 5b illustrates the DSC curves of [(CH_3_)_2_NH_2_][Mg(HCOO)_3_] with anomalies at 261–264 K at the cooling and heating rate of 5 K/min. This is consistent with the transition temperatures of 258–263 K reported by Pato-Doldan et al. [35] and 259–267 K reported by Asaji et al. [36]. The enthalpy ∆H was determined to be 2.596–2.723 kJ·mol^−1^, and the entropy ΔS was determined to be 9.8–10.4 J·mol^−1^·K^−1^. N was determined to be 3.25–3.49, indicating a 3-fold order–disorder model for the dimethylammonium cation. Asaji et al. reported similar values of ∆H, 2.7 ± 0.2 kJ·mol^−1^ and ΔS, 10 ± 1 J·mol^−1^·K^−1^ [36].

### 3.4. DMA

**Figure 6:**
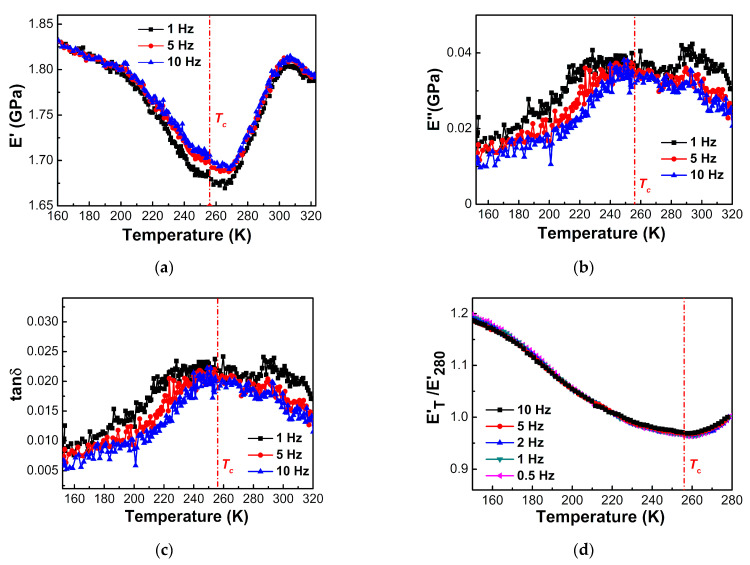

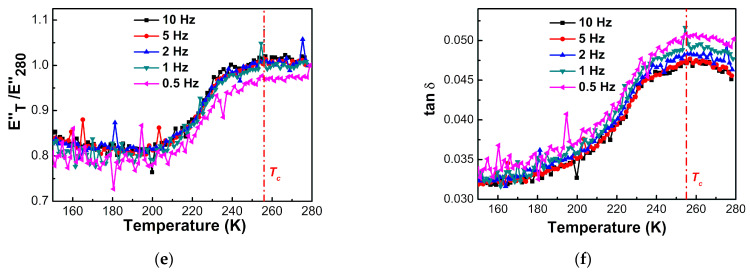
Temperature dependences of (**a**) storage modulus E’, (**b**) loss modulus E’’, (**c**) loss factor tanδ of [NH_4_][Mg(HCOO)_3_] single crystals, (**d**) normalized storage modulus E’_T_/E’_280_, (**e**) normalized loss modulus E’’_T_/E’’_298_ and (**f**) loss factor tanδ of [NH_4_][Mg(HCOO)_3_] pellet determined by DMA during heating at the rate of 2 K/min. The vertical dash-dotted line indicates the ferroelectric transition temperature of 256 K.

DMA was used to determine the complex modulus of the viscoelastic materials. The real part is the storage modulus E’, the imaginary part is the loss modulus E’’, and E’’/E’ is the loss factor tanδ. The temperature dependence of the storage modulus E’, loss modulus E’’ and loss factor tanδ of [NH_4_][Mg(HCOO)_3_] single crystals are exhibited in Figure 6a–c, and the temperature dependences of the normalized storage modulus E’_T_/E’_280_ (i.e., the ratio of the storage modulus at temperature T to that at 280 K), normalized loss modulus E’’_T_/E’’_298_ (i.e., the ratio of the loss modulus at temperature T to that at 280 K) and loss factor tanδ of the [NH_4_][Mg(HCOO)_3_] pellet are illustrated in Figure 6d–f. With the increase in temperature, the storage modulus of the single crystals and the normalized storage modulus of the pellet gradually decreased, reaching the minimum near the ferroelectric transition temperature of 256 K, and then gradually increased. With the increase in temperature, the loss modulus and loss factor of the [NH_4_][Mg(HCOO)_3_] single crystals and the normalized loss modulus and loss factor of the [NH_4_][Mg(HCOO)_3_] pellet gradually increased, reaching the maximum near 256 K, and then slightly decreased. The peaks of loss modulus, normalized loss modulus and loss factor are abroad, and they indicate weak frequency dependence.

A broad dielectric anomaly was reported in the temperature dependences of the dielectric constant for [NH_4_][Mg(HCOO)_3_], and the peak position demonstrated slight frequency dependence [15]. The resonant ultrasound spectroscopy (RUS) study of the elastic properties and acoustic dissipation associated with the disorder–order ferroelectric transition in [NH_4_][Zn(HCOO)_3_] exhibited that, with the increase in temperature, the elastic moduli, which were proportional to the square of the resonant frequencies, gradually decreased. There was also a marked change in the rate of decrease near the ferroelectric transition temperature of 192 K, and acoustic dissipation gradually increased with a peak near 192 K [16]. The Brillouin scattering (BS) study of the elastic properties and acoustic dissipation associated with the ferroelectric transition in [NH_4_][M(HCOO)_3_] (M = Mn, Zn) displayed that, with the increase in temperature, the frequency shift for the longitudinal and transverse acoustic phonons propagating along the *x* axis gradually decreased, and anomalies occurred near the ferroelectric transition temperature, with an estimated relaxation time around 4.6 × 10^−13^ s [22].

**Figure 7:**
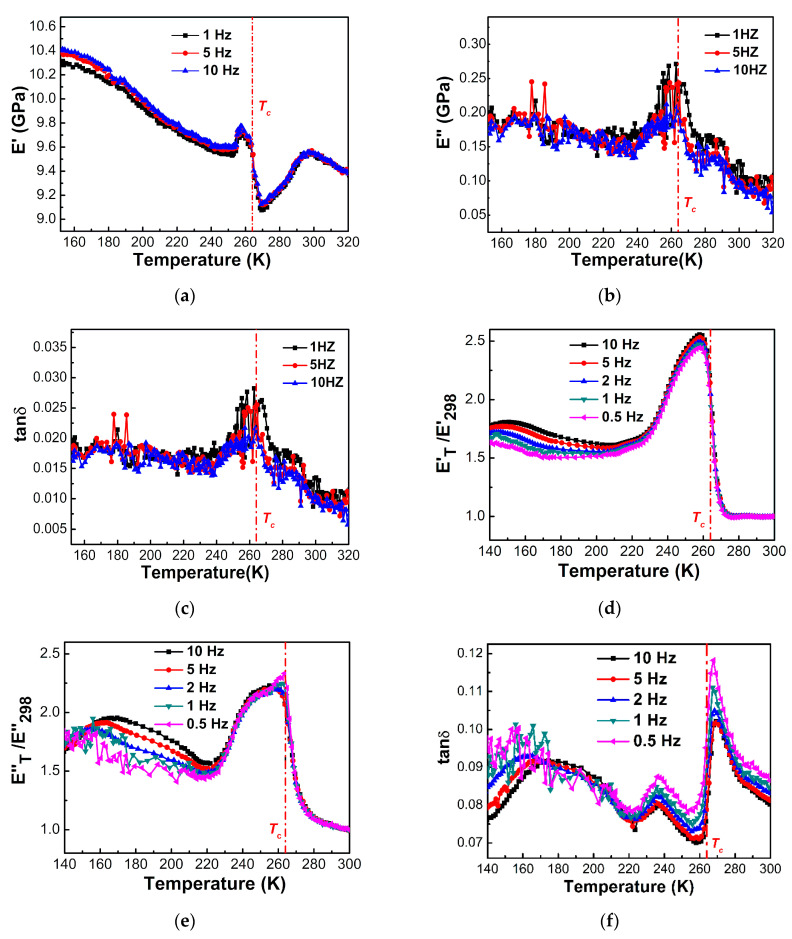
Temperature dependences of (**a**) storage modulus E’, (**b**) loss modulus E’’, (**c**) loss factor tanδ of [(CH_3_)_2_NH_2_][Mg(HCOO)_3_] single crystals, (**d**) normalized storage modulus E’_T_/E’_298_, (**e**) normalized loss modulus E’’_T_/E’’_298_ and (**f**) loss factor tanδ of [(CH_3_)_2_NH_2_][Mg(HCOO)_3_] pellet determined by DMA during heating at the rate of 2 K/min. The vertical dash-dotted line indicates the ferroelectric transition temperature of 264 K.

Figure 7 illustrates the temperature dependence of storage modulus E’, loss modulus E’’ and loss factor tanδ of the [(CH_3_)_2_NH_2_][Mg(HCOO)_3_] single crystals, and the normalized storage modulus E’_T_/E’_298_ (i.e., the ratio of the storage modulus at temperature T to that at 298 K), normalized loss modulus E’’_T_/E’’_298_ (i.e., the ratio of the loss modulus at temperature T to that at 298 K) and loss factor tanδ of the [(CH_3_)_2_NH_2_][Mg(HCOO)_3_] pellet. Near the ferroelectric transition temperature of 264 K, the storage modulus of the single crystals and the normalized loss modulus of the pellet reached the minimum, and the loss modulus and loss factor of the [(CH_3_)_2_NH_2_][Mg(HCOO)_3_] single crystals and the normalized loss modulus and loss factor of the [(CH_3_)_2_NH_2_][Mg(HCOO)_3_] pellet reached the maximum. [(CH_3_)_2_NH_2_][Mg(HCOO)_3_] displays much narrower peaks of loss modulus, normalized loss modulus and loss factor near the ferroelectric transition temperature compared with [NH_4_][Mg(HCOO)_3_]. The peak temperature tends to be independent of frequency, and the peak height tends to decrease with the increase in frequency, indicating the features of the first-order phase transition. The elastic anomalies and energy loss related to the ferroelectric transition in [(CH_3_)_2_NH_2_][Mg(HCOO)_3_] were similar to those reported for [(CH_3_)_2_NH_2_][Mn(HCOO)_3_] and [(CH_3_)_2_NH_2_][Fe(HCOO)_3_] investigated by DMA [39,53].

Asaji et al. reported that the ferroelectric transition in [(CH_3_)_2_NH_2_][Mg(HCOO)_3_] was first order, and it was associated with the freezing of the 120° reorientation of the dimethylammonium ion around the *c*-*c* axis through the two methyl groups of the cation, which were non-equivalent below the ferroelectric transition temperature of 267 K [36]. The strong frequency dependence of the dielectric constant and the dielectric loss of [(CH_3_)_2_NH_2_][Mg(HCOO)_3_] were reported, and the peak temperature increased at higher frequencies, indicating a dielectric relaxation mechanism [35]. The RUS study of the elastic properties and acoustic dissipation associated with the disorder–order ferroelectric transition in [(CH_3_)_2_NH_2_][M(HCOO)_3_] (M = Mn, Co, Ni) exhibited a broadening of the resonant peaks above the ferroelectric transition temperature and kinks in the temperature dependence of the resonant frequencies, which are proportional to elastic moduli, near the ferroelectric transition temperature of 185 K (M = Mn), 165 K (M = Co) and 180 K (M = Ni), respectively [40,41].

**Figure 8:**
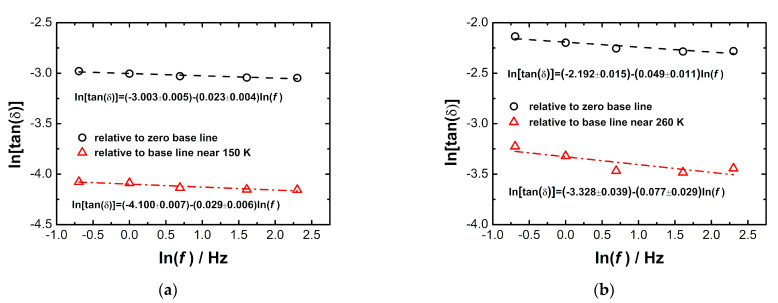
Double logarithmic plot ln(tanδ) vs. ln(f) for the peak in the temperature dependence of tanδ near 260 K in [NH_4_][Mg(HCOO)_3_] (**a**) and near 270 K in [(CH_3_)_2_NH_2_][Mg(HCOO)_3_] (**b**).

Figure 8 demonstrates a double logarithmic plot of the frequency dependence of loss factor peak height, ln(tanδ) vs. ln(f), for the peak in the temperature dependence of tanδ near 256 K for [NH_4_][Mg(HCOO)_3_], and near 264 K for [(CH_3_)_2_NH_2_][Mg(HCOO)_3_]. Two fitting processes were used to determine the values of tanδ. In the first case, the values are relative to the zero base line. In the second case, the values are relative to the base line near 150 K for [NH_4_][Mg(HCOO)_3_] and near 260 K for [(CH_3_)_2_NH_2_][Mg(HCOO)_3_]. The decay is consistent with the power law tanδ = Af^n^, where n is between −0.029 and −0.023 for [NH_4_][Mg(HCOO)_3_] and between −0.077 and −0.049 for [(CH_3_)_2_NH_2_][Mg(HCOO)_3_]. Similar features were reported for [(CH_3_)_2_NH_2_][Mn(HCOO)_3_] with n between −0.382 and −0.078 [39].

The elastic anomalies and energy loss associated with the ferroelectric transitions were detected using BS at 0–30 GHz, RUS at 0.1–2.0 MHz and DMA at 0.1–10 Hz. This suggests that multiple relaxation processes may be involved with different relaxation times, e.g., around 10^−12^ s detected by BS, around 10^−6^ s detected by RUS and around 1 s detected by DMA.

## 4. Conclusions

DMA studies of the elastic properties and energy loss of multiferroic MOFs, [NH_4_][Mg(HCOO)_3_] and [(CH_3_)_2_NH_2_][Mg(HCOO)_3_], at low frequencies of 0.5 Hz to 10 Hz between 140 K and 320 K, illustrated the minimum in storage modulus and the maximum in loss modulus and loss factor near the ferroelectric transition temperatures of 256 K and 264 K, respectively. For [NH_4_][Mg(HCOO)_3_], the peaks of the loss modulus and loss factor were broad, and they displayed weak frequency dependence. [(CH_3_)_2_NH_2_][Mg(HCOO)_3_] showed much narrower peaks of loss modulus and loss factor. The peak temperature was independent of frequency, and the peak height decreased at higher frequencies, indicating the features of the first-order phase transition. The frequency dependence of the loss factor peak height was consistent with the power law tanδ = Af^n^, where n was between −0.029 and −0.023 for [NH_4_][Mg(HCOO)_3_] and between −0.077 and −0.049 for [(CH_3_)_2_NH_2_][Mg(HCOO)_3_]. The elastic anomalies and energy loss in [NH_4_][Mg(HCOO)_3_] near 256 K are due to the second-order paraelectric to ferroelectric phase transition triggered by the disorder–order transition of the ammonium cations and their displacement within the framework channels, accompanied by the structural phase transition from the non-polar hexagonal space group *P*6_3_22 to the polar hexagonal space group *P*6_3_. The elastic anomalies and energy loss in [(CH_3_)_2_NH_2_][Mg(HCOO)_3_] near 264 K are due to the first-order paraelectric to ferroelectric phase transitions triggered by the disorder–order transitions of alkylammonium cations located in the framework cavities, accompanied by the structural phase transition from rhombohedral space group *R*$\bar{3}$c to monoclinic space group *Cc*. The elastic anomalies in [NH_4_][Mg(HCOO)_3_] and [(CH_3_)_2_NH_2_][Mg(HCOO)_3_] showed strong coupling of ferroelasticity with ferroelectricity. Multiple relaxation processes may be involved with different relaxation times, e.g., around 10^−12^ s detected by BS, around 10^−6^ s detected by RUS and around 1 s detected by DMA. Structure plays an important role in the elastic properties of MOFs. The study of the elastic anomalies and energy loss related to ferroelectric transitions is important for the development of multiferroic MOFs with high strength, ferroelectric transition temperature near room temperature, strong coupling of ferroelasticity, ferroelectricity and ferromagnetism for use in actuators, magnetic and electric devices.

## Data Availability

The data presented in this study are available on request from the corresponding authors. The data are not publicly available due to privacy reasons.
